# Sport-Specific Capacity to Use Elastic Energy in the Patellar and Achilles Tendons of Elite Athletes

**DOI:** 10.3389/fphys.2017.00132

**Published:** 2017-03-13

**Authors:** Hans-Peter Wiesinger, Florian Rieder, Alexander Kösters, Erich Müller, Olivier R. Seynnes

**Affiliations:** ^1^Department of Sport Science and Kinesiology, University of SalzburgSalzburg, Austria; ^2^Institute of Physical Medicine and Rehabilitation, Paracelsus Medical UniversitySalzburg, Austria; ^3^Department of Physical Performance, Norwegian School of Sport SciencesOslo, Norway

**Keywords:** strain energy, hysteresis, strain energy recovery, area ratio, running, jumping, swimming

## Abstract

**Introduction:** During running and jumping activities, elastic energy is utilized to enhance muscle mechanical output and efficiency. However, training-induced variations in tendon spring-like properties remain under-investigated. The present work extends earlier findings on sport-specific profiles of tendon stiffness and cross-sectional area to examine whether years of distinct loading patterns are reflected by tendons' ability to store and return energy.

**Methods:**Ultrasound scans were performed to examine the morphological features of knee extensor and plantar flexor muscle-tendon units in elite ski jumpers, distance runners, water polo players, and sedentary controls. Tendon strain energy and hysteresis were measured with combined motion capture, ultrasonography, and dynamometry.

**Results:** Apart from the fractional muscle-to-tendon cross-sectional area ratio being lower in the knee extensors of ski jumpers (−31%) and runners (−33%) than in water polo players, no difference in the considered muscle-tendon unit morphological features was observed between groups. Similarly, no significant difference in tendon energy storage or energy return was detected between groups. In contrast, hysteresis was lower in the patellar tendon of ski jumpers (−33%) and runners (−30%) compared to controls, with a similar trend for the Achilles tendon (significant interaction effect and large effect sizes η^2^ = 0.2). Normalized to body mass, the recovered strain energy of the patellar tendon was ~50% higher in ski jumpers than in water polo players and controls. For the Achilles tendon, recovered strain energy was ~40% higher in ski jumpers and runners than in controls.

**Discussion:** Advantageous mechanical properties related to tendon spring-like function are observed in elite athletes whose sport require effective utilization of elastic energy. However, the mechanisms underpinning the better tendon capacity of some athletes to retain elastic energy could not be ascribed to intrinsic or morphological features of the lower limb muscle-tendon unit.

## Introduction

The utilization of tendon elastic energy is essential in various physical tasks, in particular to minimize the energetic cost of muscular contraction or to amplify the power output of the muscle-tendon unit (MTU). Hence, different tendons with particular sets of mechanical properties may influence MTU behavior and, ultimately, mechanical output and muscular efficiency (Wilson and Lichtwark, 2011). In fact, Ker et al. (1988) had proposed a relation between the design of various mammalian tendons and their role in effective force transmission or in elastic energy saving. However, the relevance of this theory to the context of human tendon properties being tuned—via repair and remodeling—with training is largely unexplored.

The different tendon cross-sectional areas (CSA) found between athletes (Rosager et al., 2002; Kongsgaard et al., 2005) or the different tendon stiffness measured in athletes specialized in different types of running (Arampatzis et al., 2007) seem to support the hypothesis of functionally driven tendon adaptations. However, such studies have thus far focused on certain variables (e.g., tendon morphology) or certain activities (e.g., running), preventing a broader interpretation of training-induced tendon adaptations. Moreover, only few studies have investigated the influence of training on variables related to the capacity to store and release elastic energy *in vivo* (see Wiesinger et al., 2015 for review). Decreases in hysteresis measured following plyometric or resistance training indicate that these forms of exercise are effective in reducing the energy lost during the recoil of the patellar (PT) (Reeves et al., 2003b) and Achilles tendons (AT) (Kubo et al., 2002, 2012; Fouré et al., 2010). However, beyond these interesting findings and the increase in stiffness typically observed after such interventions, the long-term optimization of tendon properties to store and return energy remains under-investigated.

In the present study, we conducted an exhaustive assessment of *in vivo* morphological and mechanical properties of the PT and AT in elite athletes, specialized in activities requiring different patterns of loading. The training of ski jumpers typically includes forceful, explosive contractions. Distance runners engage in high volumes of cyclic loading and the lower-limbs muscles of water polo players have to perform considerable amounts of work because of the reduced possibility to use elastic energy in the water element. The recruitment of these athletes was based on the different predominant roles played by their tendons and the fact that they had years of practice and adaptation. In an initial report (Wiesinger et al., 2016), we observed similar values of absolute stiffness between groups, although athletes involved in activities requiring power amplification (ski jumpers) had stiffer tendons than untrained controls relative to their body mass. Interestingly, stiffness was not systematically associated with a commensurate tendon CSA, suggesting a dual mechanism of adaptation driven by functional demand and structural integrity. Whether this dissociation between tendon morphological and mechanical features pertains to energy storage capacity is unknown.

The present article addresses the question of the utilization of elastic energy. Is the tendon capacity to store and release energy influenced by years of differentiated training and loading patterns? The main purpose was to investigate whether the tendons of athletes requiring larger utilization of elastic energy (ski jumpers and runners) would have optimized properties when compared to other types of athletes (water polo players) or untrained controls.

Considering the earlier findings described above (Wiesinger et al., 2016) and the physical link between energy storage and tendon stiffness, we expected to find similar strain energy values across groups. Likewise, we did not expect the tendon capacity to store energy to be consistently reflected by morphological features of the MTU (e.g., muscle-to-tendon area ratio). A lower hysteresis and higher elastic energy return were hypothesized in ski jumpers and runners whereas we expected the tendons of water polo players to display hysteresis levels comparable to that of untrained controls.

## Materials and methods

### Subjects

The experiment was conducted on 39 male subjects (18–40-year old) previously recruited for a companion paper published elsewhere (Wiesinger et al., 2016). Three groups of elite athletes, 10 ski jumpers (Austrian and German National Team), 10 highly trained endurance runners and 9 water polo players (Austrian Champion) were compared to each other and to 10 non-physically active controls (Table 1). Subjects were textually and/or verbally contacted, fully informed about the nature, aim, risks, and benefits of the experiment. Potential athletes or non-active controls were excluded from the study if they reported any knee or ankle injuries, cardiovascular disorders, respiratory and neuromuscular diseases, history of diabetes, or ergogenic drug abuse. Runners, water polo players and control subjects did not have any prior history of resistance training. Additionally, controls were not engaged in regular (>1/week), strenuous physical activity. Ski jumpers had a training experience of 10-to-18 years and were tested in the preconditioning phase, in which training consisted of 8–10 sessions per week, with a focus on explosive resistance training. Their typical training routine required high-intensity contractions, with low number of repetitions at high levels of resistance and explosive jumps. The training load of endurance runners involved 60–100 km/week, corresponding to ~31.400 loading cycles of the PT and AT per week (Lichtwark et al., 2013). This group had been training for 5-to-13 years. Water polo athletes had trained in all-deep water pools 4-to-6 times (2 h) a week for 4-to-15 years. These athletes did not take part to any land-based exercise. Control subjects consisted of employees from the University of Salzburg and from the local community of the City of Salzburg. All subjects provided a written informed consent in accordance with the Declaration of Helsinki and the Ethics Committee of the University of Salzburg approved the research protocol.

**Table 1:** **Anthropometric characteristics and training experience of participants**.

	**Ski jumper**	**Runner**	**Water polo**	**Control**	***F*****_(3;35)_**	***P*****-value**	**η^2^-value**
Age (yrs)	22.2 ± 2.9^†††^^***^	31.5 ± 4.6	24.2 ± 3.2^†††^^***^	31.0 ± 5.1	13.08	<0.001	0.53
Body height (cm)	176.3 ± 4.5	180.9 ± 8.2	182.4 ± 6.5	182.9 ± 7.2	1.96	0.140	0.14
Body mass (kg)	64.3 ± 3.9^†^^§§§^^***^	72.8 ± 7.6^§^	84.3 ± 10.8	83.9 ± 12.3	10.72	<0.001	0.48
PT MA (cm)	3.39 ± 0.15	3.43 ± 0.18	3.31 ± 0.18	3.41 ± 0.18	0.84	0.482	0.07
AT MA (cm)	5.32 ± 0.34	5.37 ± 0.72	5.81 ± 0.36	5.73 ± 0.64	2.00	0.132	0.15
Training experience (yrs)	13.9 ± 2.2	7.8 ± 2.6	9.0 ± 3.5	0.0 ± 0.0	56.62	<0.001	0.83

*Values are expressed as mean ± SD. PT, patellar tendon; AT, Achilles tendon; MA, moment arm; BMI, body mass index*;

† *P < 0.05*;

††† *P < 0.001 compared with runners*;

§ *P < 0.05*;

§§§ *P < 0.001 compared with water polo players*;

*** *P < 0.001 compared with controls (Wiesinger et al., 2016)*.

### Experimental design

**Figure 1:**
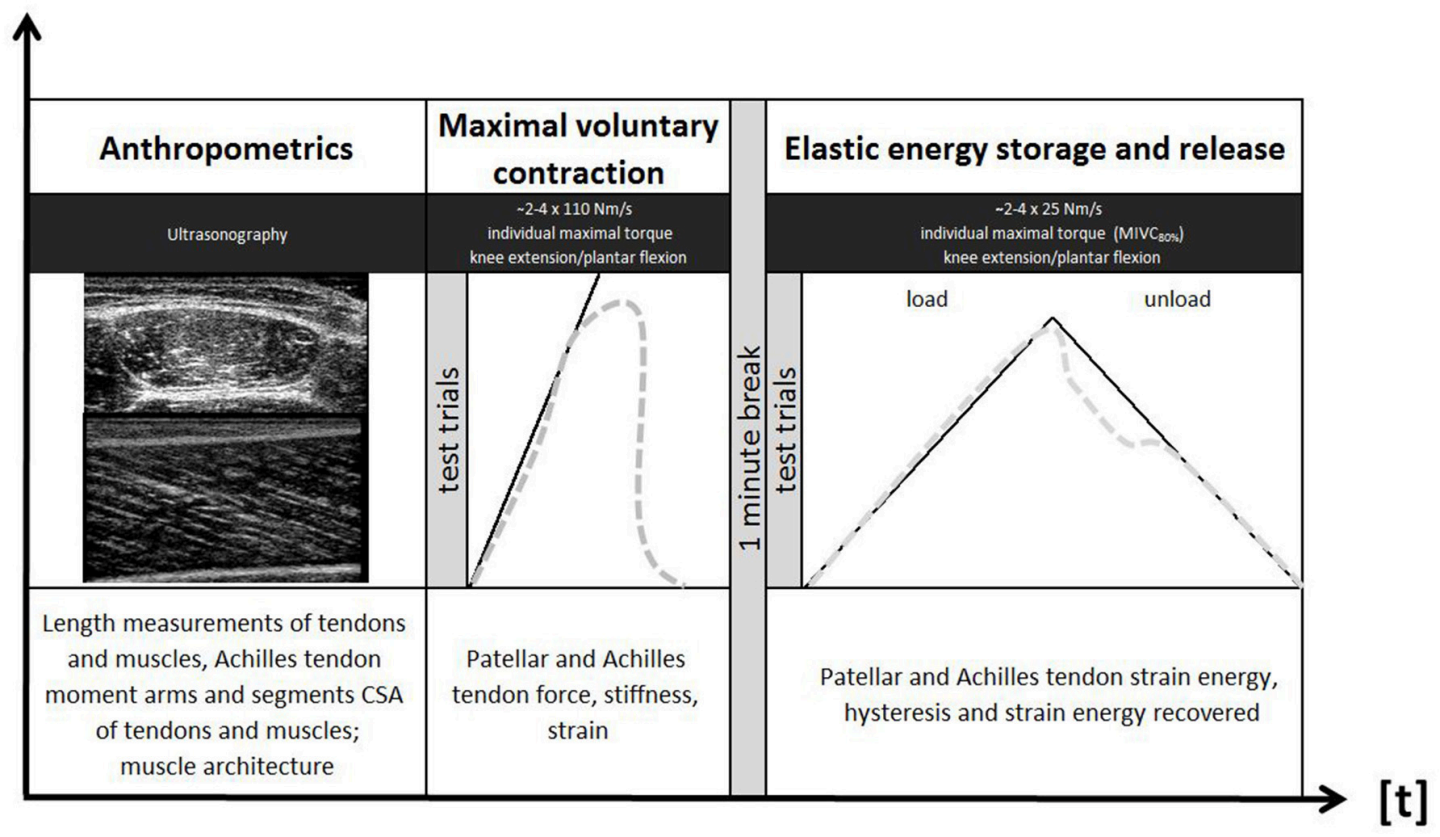
**Illustration of the experimental design**.

Figure 1 summarizes the experimental design of this study. Leg dominance was determined (Büsch et al., 2009) and tests were conducted according to a block randomization on either leg. Athletes were asked to refrain from vigorous exercise (e.g., resistance training, running, jumping) during 24 h before the testing sessions. Ski jumpers and runners were tested outside their competition period and water polo players were tested at least 3 days following their last competition event. The inter-day reliability of muscle CSA, tendon strain energy and hysteresis measurement was assessed in a subgroup (control subjects) with an additional testing session, 3-to-5 days (± 2 h of day time) following the first one. Individual target torque levels were held constant over the remainder of the experiment.

### Muscle anatomical CSA and architecture

Muscle CSA was imaged via real-time ultrasonography in gastrocnemius medialis (GM), vastus lateralis (VL), and rectus femoris (RF) and measurements of pennation angle and fascicle length were obtained in the former two. Ultrasound scans were performed over the mid-belly of the GM muscle and at 40% of the femur length for the VL and RF muscles (Rieder et al., 2015b). Measurements were conducted by the same investigator (HW) on resting subjects.

Muscle CSA was imaged via panoramic scans (12L-SC, 8.0- to 13.0 MHz transducer, LOGIQ e Ultrasound—BT12, General Electric Company), while pennation angle and fascicle length were measured from regular transverse ultrasound scans (linear array transducer 5 cm, LA523, 10- to 15-MHz transducer, MyLab25, Easote, Genoa, Italy). All images were analyzed offline (ImageJ, Institutes of Health, Bethesda, MD, 172 USA. http://imagej.nih.gov/ij/). Fascicles were manually outlined. When fascicle length exceeded the ultrasound field of view, a linear approximation was applied (Muraoka et al., 2001; Rieder et al., 2015a). The angle relative to the deeper aponeurosis was defined as pennation angle. The average of two measurements was used for further analysis. The good reliability of ultrasound-based architecture measurement has been shown previously (Sipilä and Suominen, 1993; Narici et al., 1996; Seynnes et al., 2008). Panoramic scans used for muscle CSA in this experiment also showed satisfactory repeatability, with intraclass correlation coefficients ranging from 0.95 to 0.99 and typical errors of 2.7% for the vastus laterialis and 2.1% for the gastrocnemius medialis.

### Morphology of the patellar and achilles tendons

Patellar and Achilles tendon length were measured from longitudinal panoramic ultrasound scans, in the same position in which force and tendon mechanical properties were obtained (see below). Thus, the PT length was imaged at a knee joint angle of 90° (0° corresponding to full extension), as the distance between the patellar apex and the tibial tuberosity. Achilles tendon length was defined as the distance between the calcaneal insertion and the gastrocnemius myotendinous junction, obtained with the knee and hip fully extended and the ankle joint at 90°.

Measurements of tendon CSA were obtained from transverse scans. Patellar tendon CSAs were outlined from the proximal, tendon mid-length and distal areas and an average value was used for further analysis. The AT CSA was measured at the distal end of the soleus muscle, corresponding to a tendon region where this parameter can be obtained reliably (Stenroth et al., 2012). ImageJ was also used for offline analysis of all tendon morphological measurements.

### Maximal voluntary contraction

After a standardized, 10-min supervised warm-up protocol on a stationary ergometer (Heinz Kettler GmbH and Co. KG, Ense-Parsit, Germany) at a sub-maximal intensity of 1.5 W/kg and a cadence of ~70 rpm, lower leg strength measurements were performed on a rigid isokinetic dynamometer (IsoMed 2000 D&R Ferstl GmbH, Hemau, Germany).

Patellar and Achilles tendon force and maximal strain were recorded during two separate isometric ramp contractions at a constant loading rate of 110 Nm/s to maximal exertion, to enable effective analysis of ultrasound recordings. Tendon forces were calculated offline, taking antagonist muscle activation and individual moment arm lengths of the knee and ankle joints in consideration (Wiesinger et al., 2016).

For each test, the rotational center of the dynamometer arm was carefully aligned with that of the tested joint and sliding of the subjects was minimized with straps and pads over the shoulders, hip joint and thighs. Subjects received detailed instructions and several practice trials before each testing task. Strong verbal encouragements and visual feedback were provided during all contractions. Trials with the highest torque were retained for further analysis.

### Elastic energy storage and release in the patellar and achilles tendons

Patellar and Achilles tendon capacity to utilize elastic energy storage and release was assessed in the same positions in which subjects performed maximal voluntary contractions. Tendon forces were recorded simultaneously with ultrasound scans showing the displacement of tendon insertions. Artefactual joint movements were accounted for by setting a high-speed video camera (JVC GC-PX100BEU at 200 Hz) perpendicular to the sagittal plane, capturing reflective markers placed on the dynamometer footplate, over the subjects' calcaneous bone and the ultrasound probe (linear array transducer 5 cm, LA523, 10- to 15-MHz transducer, MyLab25, Easote, Genoa, Italy).

**Figure 2:**
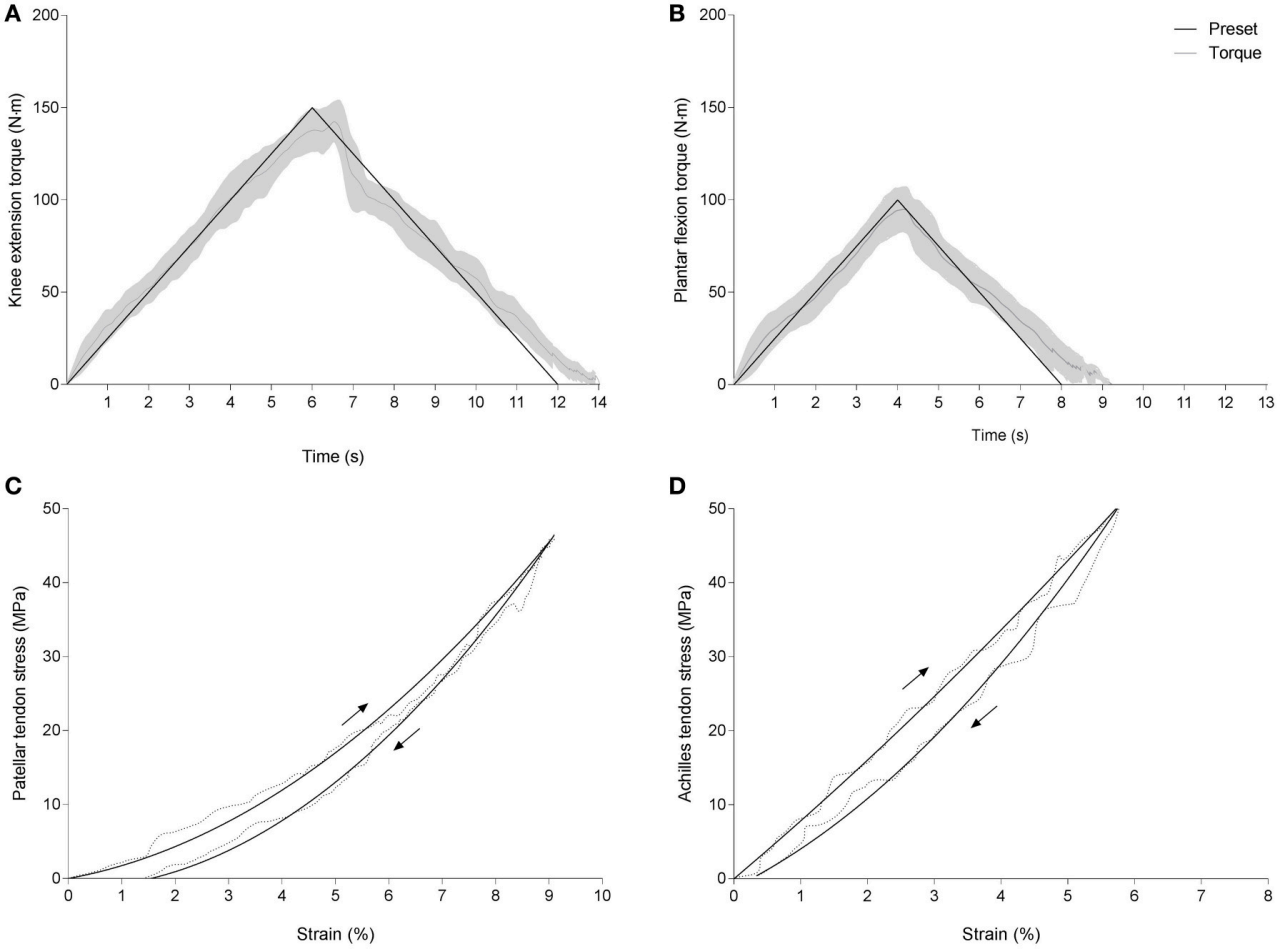
**Between group comparison of patellar and Achilles tendon spring efficiency**. Plots show knee extension and plantar flexion torque development during predefined hysteresis trials shown among the subjects and plotted against time **(A,B)** and typical stress-strain curves from one subject **(C,D)**. Dotted lines in **(C,D)** are raw data and solid lines are based on least squares fit method with constraints.

Tendon strain energy, hysteresis and strain energy recovered were obtained from ramp contractions with symmetrical isometric loading and unloading ramps at a predefined rate of 25 Nm/s (see Figures 2A,B). Different loading rates were empirically chosen for the ramp contraction tests, to account for the double necessity to load the tendon slowly enough for maximal strain measurements and fast enough to enable appropriate motor control. Indeed, unloading ramps appeared more demanding at slower paces and at maximal force levels, yielding insufficient target torque matching. Accordingly, a target torque level of 80% of individual maximal isometric voluntary contraction torque level (MIVC_80%_) was used for this test. Subjects were familiarized with these tasks by using a visual feedback displaying the torque produced and targeted. Familiarization trials also served as a conditioning routine preceding each test. Familiarization and test trials were repeated until the subjects were able to match the displayed loading patterns satisfactorily (from visual inspection). Test trials were separated by a pause of 30 s and loading cycles where the moment deviated from individual MIVC by more than 5% were discarded. The mean difference between target (25 Nm/s) and actual loading rates ranged −3.4 to +2.1 Nm/s (~24.6 Nm/s) for the loading phase and −1.9 to +3.5 Nm/s (~–24.3 Nm/s) for the unloading phase. Patellar and Achilles tendon force-deformation and stress-strain data were subsequently fitted using least squares approximation within constraints based on minimal and maximal stress (lsqlin function, see Figures 2C,D). Data sets retained for further analysis presented correlation coefficients (*R*^2^) higher than 0.97 and 0.92, respectively, for the loading and unloading phases.

Hysteresis values were calculated offline as the percent difference between the areas under stress-strain loading and unloading curves (MIVC_80%_). An average value obtained from two trials was used for each subject. Patellar and Achilles tendon strain energy was determined as the largest area under the ascending force-deformation curves of loading phase. The maximum strain energy recovered, measured as the largest area under the descending curve. Strain energy and strain energy recovered were normalized to body mass (Shadwick, 1990) to account for the daily loading of lower limb tendons and the physical activity patterns of the different groups. Furthermore, normalized values were included to give a functional perspective to the calculated tendon mechanical properties. The intraday reliability of tendon hysteresis measured in PT and AT yielded an intraclass correlation coefficient of 0.80 in both cases, with typical errors of 1.92 and 2.14%, respectively. Reliability of elastic strain energy calculations was also high, with intraclass correlation coefficients of 0.93 and 0.91, and typical errors of 0.67 J and 1.92 J, respectively, for the PT and AT. Measurements of strain energy recovered yielded an intraclass correlation coefficients of 0.92 and 0.90, with a typical error of 0.01 and 0.02 J/kg, respectively, for the PT and AT.

The dimensionless fiber length factor, $\hat{L}$, (ratio of fascicle length to tendon extension) (Ker et al., 1988; Zajac, 1989; Shadwick, 1990) was calculated to characterize the MTU suitability for effective force transmission and muscular work or for spring-like function and efficiency. Maximal tendon extension was measured under MIVC trials at the highest PT and AT stress common to all subjects (PT 41 MPa; AT 33 MPa), while muscle fascicle length and tendon length were examined at rest. In other words, the parameter $\hat{L}$ is a morphological expression of strain energy storage capacity under maximal contraction. Lower values of $\hat{L}$ (<2, *in vitro*) have been measured in mammalians whose MTU requires daily spring-like function, with relatively long and thin tendons (Ker et al., 1988).

Furthermore, ratios of muscle to tendon areas (A_m_/A_t_) were calculated. Since muscle CSA could not be measured in the entire quadriceps and triceps surae with ultrasonography, this parameter was obtained as the ratio of muscle CSA to fractional tendon CSA. The latter was calculated as the fraction of tendon CSA corresponding to the proportion of muscle CSA relative to its muscle group (Akima et al., 1995; Fukunaga et al., 1996).

### Statistics

The statistical analysis was conducted using SPSS Statistics V.22.0 (SPSS Inc., Chicago, Illinois, USA). A Kolmogorov–Smirnov test was used on all variables to test their normality of distribution. The significance of between-groups differences in each variable was tested with a one-way ANOVA including a Tukey-type correction in case of significant between-group effects to account for multiple comparisons. The effect size (η^2^) was defined as small for η^2^ > 0.01, medium for η^2^ > 0.06, and large for η^2^ > 0.14 (Cohen, 1988). F- or Q-statistics and *P*-values are reported so that the relative degree of significance can be assessed in all cases. Figures were created using the GraphPad Prism 7.00 (GraphPad Software Inc; La Jolla; USA). Data are presented as means and standard deviations. The critical significance level was established at *P* < 0.05 for all statistical tests.

## Results

The physical characteristics and training experience of the subjects are reported in Table 1.

### Muscle anatomical CSA and architecture

Muscle CSA did not differ significantly between groups [main effects: RF: *F*_(3;35)_ = 1.09, *P* = 0.368, η^2^ = 0.09; VL: *F*_(3;35)_ = 1.62, *P* = 0.203, η^2^ = 0.12; GM: *F*_(3;35)_ = 1.71, *P* = 0.183, η^2^ = 0.13]. The pennation angle of the vastus lateralis [*Q*_(4;35)_ = 3.38, *P* = 0.009, η^2^ = 0.36 and *Q*_(4;35)_ = 3.12, *P* = 0.018, η^2^ = 0.28] and gastrocnemius medialis [*Q*_(4;35)_ = 2.98, *P* = 0.026, η^2^ = 0.35 and *Q*_(4;35)_ = 2.76, *P* = 0.043, η^2^ = 0.26] were higher in ski jumpers and runners than in the control group [main effects: *F*_(3;35)_ = 5.20, *P* = 0.004, η^2^ = 0.31 and *F*_(3;35)_ = 3.19, *P* = 0.016, η^2^ = 0.25, respectively]. No other differences were found for this variable. Muscle fascicles of the vastus lateralis were significantly longer in water polo athletes than in runners [+27%, *Q*_(4;35)_ = 2.71, *P* = 0.048, η^2^ = 0.02] but differences between other groups did not reach significance [main effect: *F*_(3;35)_ = 2.66, *P* = 0.064, η^2^ = 0.19]. In the gastrocnemius medialis, fascicle length was similar across all groups [main effect: *F*_(3;35)_ = 0.62, *P* = 0.608, η^2^ = 0.05].

### Patellar and achilles tendon properties

Compared to controls, hysteresis measured at MIVC_80%_ was significantly lower in the PT of ski jumpers [–33%, *Q*_(4;35)_ = 3.19, *P* = 0.015, η^2^ = 0.27] and runners [–30%, *Q*_(4;35)_ = 2.93, *P* = 0.029, η^2^ = 0.40] but not in water polo athletes [–14%, *Q*_(4;35)_ = 1.33, *P* = 0.551, η^2^ = 0.11]. None of the group differences observed in AT hysteresis reached statistical significance in tests performed at MIVC_80%_, despite a trend toward lower values in ski jumpers [*Q*_(4;35)_ = 2.52, *P* = 0.074, η^2^ = 0.21] and runners [*Q*_(4;35)_ = 2.62, *P* = 0.060, η^2^ = 0.32] compared to controls [main effect: *F*_(3;35)_ = 3.86, *P* = 0.017, η^2^ = 0.25].

**Figure 3:**
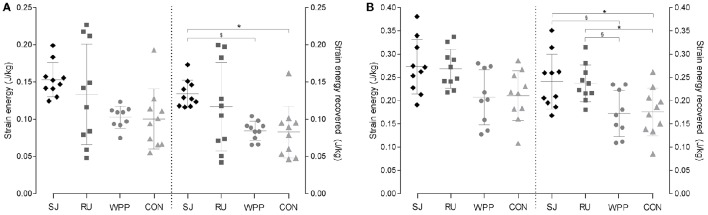
**Normalized elastic strain energy and strain energy recovered from the patellar (A)** and Achilles tendon **(B)**. Scatter plot with mean ± standard deviation for ski jumpers (SJ), runners (RU), water polo player (WPP), and controls (CON) are shown. ^§^*P* < 0.05 compared with water polo players; ^*^P < 0.05 compared with controls.

According to *post hoc* comparisons, PT and AT capacities to store strain energy was similar between groups, despite significant interaction effects when normalized to body mass (Tables 2, 3). Similarly, raw values of strain energy recovery did not differ between groups. However, after normalization to body mass, the strain energy recovered by elastic recoil was higher in the PT of ski jumpers than that of water polo athletes [+55%, *Q*_(4;35)_ = 2.72, *P* = 0.048, η^2^ = 0.72] and controls [+62%, *Q*_(4;35)_ = 3.03, *P* = 0.023, η^2^ = 0.49]. No other group differences were found. The recovered strain energy from the AT of ski jumpers and runners was higher than that of water polo players [+40%, *Q*_(4;35)_ = 2.96, *P* = 0.027, η^2^ = 0.30; +37%, *Q*_(4;35)_ = 2.79, *P* = 0.040, η^2^ = 0.37] and controls [+37% *Q*_(4;35)_ = 2.90, *P* = 0.031, η^2^ = 0.28;+35%, *Q*_(4;35)_ = 2.73, *P* = 0.046, η^2^ = 0.33], with no difference between ski jumpers and runners [*Q*_(4;35)_ = 0.17, *P* = 0.998, η^2^ < 0.01] when looking at normalized values. No other differences were found for this variable (Tables 2, 3, Figures 3A,B).

**Table 2:** **Patellar tendon elastic energy storage and release**.

	**Ski jumper**	**Runner**	**Water polo**	**Control**	***F*****_(3;35)_**	***P*****-value**	**η^2^-value**
**TENDON FORCE/PROPERTIES** (Wiesinger et al., 2016)							
Tendon force (N)	6948 ± 1235	6653 ± 676	7255 ± 1550	6733 ± 1063	0.50	<0.682	0.04
Strain (%)	8.4 ± 1.7	8.2 ± 2.3	8.9 ± 1.6	8.6 ± 1.4	0.32	0.814	0.03
**STORAGE/RECOIL**							
Hysteresis (%)	12.1 ± 5.8^*^	12.5 ± 2.6^*^	15.5 ± 3.2	18.0 ± 4.3	4.40	0.010	0.27
Strain energy (J)	9.8 ± 1.3	9.4 ± 4.0	8.6 ± 1.0	8.7 ± 4.7	0.62	0.605	0.05
Strain energy (J/kg)	0.15 ± 0.03	0.13 ± 0.07	0.10 ± 0.01	0.10 ± 0.04	3.19	0.035	0.22
Strain energy recovered (J)	8.60 ± 1.00	8.18 ± 3.47	7.24 ± 0.84	7.18 ± 4.05	0.62	0.606	0.05
Strain energy recovered (J/kg)	0.13 ± 0.02^§^^*^	0.12 ± 0.06	0.09 ± 0.01	0.08 ± 0.03	4.09	0.014	0.26
**MUSCLE-TENDON RATIOS**							
Fiber length factor ($\hat{L}$)	36.31 ± 8.57	25.59 ± 10.41	30.51 ± 8.50	29.46 ± 10.32	1.76	0.174	0.131
Area ratio (A_m_/A_t_)	43.0 ± 9.8^§§^	42.2 ± 10.7^§§^	62.5 ± 11.3	49.5 ± 10.5	7.38	0.001	0.39

*Values are mean ± SD. A_m_, cross-sectional area of the gastrocnemius muscle, A_t_, fractional Achilles tendon cross-sectional area*.

§ *P < 0.05*;

§§ *P < 0.01 compared with water polo players*;

* *P < 0.05 compared with controls*.

**Table 3:** **Achilles tendon elastic energy storage and release**.

	**Ski Jumper**	**Runner**	**Water Polo**	**Control**	***F*****_(3;35)_**	***P*****-Value**	**η^2^-Value**
**TENDON FORCE/PROPERTIES** (Wiesinger et al., 2016)							
Tendon force (N)	3639 ± 326	4340 ± 822	4133 ± 486	3900 ± 690	2.40	0.085	0.17
Strain (%)	6.5 ± 1.9	6.1 ± 1.3	6.1 ± 1.5	6.2 ± 1.5	0.12	0.945	0.01
**STORAGE/RECOIL**							
Hysteresis (%)	12.0 ± 5.6	11.8 ± 3.1	16.6 ± 4.5	17.3 ± 5.1	3.86	0.017	0.25
Strain energy (J)	17.6 ± 3.9	19.5 ± 3.5	17.8 ± 6.6	17.2 ± 2.8	0.76	0.526	0.06
Strain energy (J/kg)	0.27 ± 0.06	0.27 ± 0.04	0.21 ± 0.06	0.21 ± 0.05	4.34	0.011	0.27
Strain energy recovered (J)	15.49 ± 3.76	17.17 ± 2.85	14.76 ± 5.42	14.27 ± 2.91	1.09	0.367	0.09
Strain energy recovered (J/kg)	0.24 ± 0.06^§^^*^	0.24 ± 0.04^§^^*^	0.17 ± 0.05	0.18 ± 0.05	5.42	0.004	0.32
**MUSCLE-TENDON RATIOS**							
Fiber length factor ($\hat{L}$)	8.12 ± 2.58	7.78 ± 2	9.36 ± 4.21	8.06 ± 1.92	0.59	0.626	0.05
Area ratio (A_m_/A_t_)	97.7 ± 26.9	97.3 ± 8.3	130.1 ± 39.7	114.2 ± 37.2	2.52	0.074	0.18

*Values are mean ± SD. A_m_, combined cross-sectional area of the rectus femoris and vastus lateralis muscle, A_t_, fractional patellar tendon cross-sectional area*.

§ *P < 0.05 compared with water polo players*;

* *P < 0.05 compared with controls*.

### Fiber length factor and muscle-to-tendon area ratio

Patellar [main effect: *F*_(3;35)_ = 1.63, *P* = 0.200, η^2^ = 0.12] and Achilles tendon [main effect: *F*_(3;35)_ = 1.54, *P* = 0.222, η^2^ = 0.12] length normalized to leg length did not differ between groups (Wiesinger et al., 2016). The fiber length factor $\hat{L}$ did not differ between groups (Tables 2, 3). The ratio of VL and RF muscle to fractional PT CSA was lower in ski jumpers and runners than in water polo players [−31%, *Q*_(4;35)_ = 4.02, *P* = 0.002, η^2^ = 0.10 and −33%, *Q*_(4;35)_ = 4.19, *P* = 0.001, η^2^ = 0.12, respectively], but did not differ significantly between other groups. Similar trends were observed for the ratio of GM muscle to fractional AT CSA (main effect: *P* = 0.074) but none of the numerical differences reached significance (Tables 2, 3).

## Discussion

The aim of this comparison study was to determine whether the effects of long-term sport-specific loading patterns are reflected by different tendon capacities to store and release elastic energy. In agreement with our hypothesis, PT and AT energy storage capacities were statistically similar across groups, while the tendons of athletes requiring larger utilization of elastic energy (ski jumpers and runners) displayed a lower hysteresis than water polo players and controls. However, the mechanisms underpinning this better capacity to retain elastic energy could not be ascribed to intrinsic or morphological features of the lower limb MTU.

The variety of tendon designs (e.g., CSA) within and between mammalian species has been linked to functional constraints, opposing tendons with the main function of elastic energy utilization to tendons required to transmit force effectively. Using estimations of peak tension in tendons, Ker et al. (1988) have associated the necessity to store elastic energy with higher ratios of muscle to tendon area (A_m_/A_t_ > 75). Consistent with these observations, the higher ratios observed on average for the AT (A_m_/A_t_ = 109.3 ± 32.1) in the present experiment indicated that this tendon is more suited to work as a spring than the PT (A_m_/A_t_ = 49.0 ± 12.9). However, lower muscle-to-tendon cross-section ratios were found in the knee extensor muscles of ski jumpers and runners than in water polo players, with a similar trend (*P* = 0.074 for interaction effect) for the plantar flexors. Tendons were therefore thicker, relative to muscle CSA, in athletes whose activity requires an important use of elastic energy. These observations are congruous with the larger tendon CSA, relative to the body mass, measured in these athletes (Wiesinger et al., 2016), yet they were unexpected in athletes whose daily activity requires sufficient tendon strain and elastic energy storage to amplify power output or to reduce the energy cost of mechanical work (Alexander et al., 1982; Roberts et al., 1997). Alternatively, one could argue that A_m_/A_t_ ratio is optimal in ski jumpers and endurance runners but the similar values found in controls and, importantly, the higher ratios observed in water polo players seem at odds with the idea of a functional optimization of tendon design. Indeed, the water medium limits energy transfer within the MTUs of lower limbs, implying that thick and stiff tendons (i.e., a lower A_m_/A_t_ ratio) would be better suited to transmit force effectively in water polo players. Hence, in the context of adaptations to training, tendon CSA may be adjusted to preserve its integrity (e.g., against excessive damage) rather than to comply with requirements of elastic energy utilization.

Despite the lower A_m_/A_t_ ratio observed in some athletes, the maximal strain energy that can be stored in the AT and PT was not found different between athletes. If anything, the tendons of runners and ski jumpers tended to have higher energy storage capacities than water polo players and controls (Figure 3). This contrasting finding further suggests that mechanical and morphological properties can be uncoupled and that the spring-like function of tendons can be preserved in spite of a relatively large CSA. This interpretation is consistent with our previous observations (Wiesinger et al., 2016) indicating that differences in tendon CSA were not always mirrored by differences in stiffness. Adjustments in material properties may alter the relation between tendons CSA and deformation to uniaxial force (Reeves et al., 2003a), reflecting on their ability to store elastic energy.

As illustrated by Lichtwark and Wilson (2008) in the case of gait efficiency, a coordinated optimization of tendon mechanical properties and muscle fascicle length may be necessary to optimize lower limb function. To account for this aspect and for inter-individual differences in MTU morphology, the parameter $\hat{L}$ was introduced, which illustrates the relative importance of muscle- vs. tendon length changes. As expected, this index of energy storage capacity confirmed the better suitability of AT ($\hat{L}$ = 22.54 ± 7.66) than PT ($\hat{L}$ = 4.51 ± 1.74) to work as a spring. However, in line with the similar tendon strain energy observed between athletes, $\hat{L}$ did not differ significantly between groups. This lack of statistical difference results from the fact that fascicle length of the vastus lateralis or gastrocnemius medialis and maximal PT or AT strain were relatively homogeneous between groups. Fascicles were only found significantly longer in the gastrocnemius of water polo players in comparison to runners, which incidentally fits with the hypothesis of a higher work demand for the formers (Figure 3). Taken together, the lack of significant difference in $\hat{L}$ and in strain energy challenge the hypothesis of a systematic, training induced specialization of tendons to store energy.

Hysteresis values (~12–18%) measured here are comparable to previous *in vivo* measurements (see Finni et al., 2013 for review) and the similarities found between the PT and AT within each group are in agreement with animal (Pollock and Shadwick, 1994) and human (Maganaris, 2002) studies suggesting that energy dissipation is independent of physiological tendon function. However, when comparing groups, hysteresis was lower in the PT of ski jumpers and endurance runners than in controls, with a similar trend (large effects η^2^ > 0.2) for the AT (significant interaction effect, *P* = 0.017). These differences indicate a better energy conservation capacity in athletes whose tendons mainly work as springs. Incidentally, they match decreases in tendon hysteresis (~30%) typically observed in response to high intensity resistive training (Kubo et al., 2002, 2012; Reeves et al., 2003b; Fouré et al., 2010). With regard to these results, the lack of statistical difference in strain energy recovery between groups was surprising. Energy storage being similar between groups, we expected a higher elastic energy return to explain the lower hysteresis values. Such a discrepancy may be explained by an insufficient statistical power (see *Limitations* bellow) and further research is required to ascertain whether an optimization of energy return is related to the lower hysteresis seen in certain athletes.

In the absence of tendon damage, a lower hysteresis is generally attributable to differences in material properties. The structure and composition of the tendon seems to contribute to gross differences in mechanical properties, ultimately influencing energy storage or positional control (see Thorpe et al., 2016 for review). For instance, some non-collagenous components embedded within the extra-cellular matrix (e.g., glycoprotein lubricin) may facilitate interfibrillar sliding and reduce energy dissipation. Additionally, the ability of the tendon to recover stored energy is likely related to the typology and density of cross-links (Birch, 2007), to the presence of elastin fibers (Millesi et al., 1995) or to crimp patterns of collagen fibrils (Benjamin et al., 2008; Franchi et al., 2009). The influence of tendon composition cannot be established from the present data but *in vivo* measurements of Young's modulus performed on the same subjects did not indicate any difference between athletes (Wiesinger et al., 2016). The lack of difference in Young's modulus and the lower hysteresis measured in some athletes could indicate that tendon structure and composition influence mechanical properties related to tendon loading and unloading in different ways.

Nevertheless, the lower hysteresis found in runners and ski jumpers—but not in water polo players—is congruent with the hypothesis of functionally driven adaptation, which potentially enhances elastic energy conservation and power amplification.

In line with this hypothesis, AT energy return per unit of mass is ~40% higher in ski jumpers and runners than controls and about 50% higher in the PT of ski jumpers than in water polo players and controls (Tables 2, 3). Ultrasound imaging has shown the important role of elastic energy during running (Lichtwark and Wilson, 2006; Farris et al., 2012) and jumping tasks (Kurokawa et al., 2001). Although human muscle lacks the anatomical “catch mechanism” observed in some animals (Gronenberg, 1996), elastic energy may be stored during muscle contraction and released to amplify power production before take-off, hereby contributing to running and ski jump performance.

## Limitations

Potential limitations should be considered before concluding. Firstly, the inclusion of four groups and the relatively low sample size certainly decreased statistical power. An *a posteriori* analysis confirmed that the tests performed on hysteresis were slightly underpowered (power = 0.77). This limitation may have impacted the *post-hoc* analysis, with between-groups differences in AT hysteresis missing statistical significance despite large effect sizes (η^2^ > 0.20; Cohen, 1988).

Additionally, elastic properties of structures within the muscle (e.g., cross-bridges, actin and myosin filaments, collagen fibers, or titin) may have the potential to provide useful energy storage and recovery during certain movements (see Roberts, 2016 for review). The influence of the present findings on the MTU mechanical output is certainly impacted by our inability to measure these contributions *in vivo*.

Secondly, our measurements of muscle architectural and morphological characteristics were only based on local ultrasound scans of selected muscles. The mechanical output of the MTU may have been better reflected by measurements of physiological CSA, performed over whole muscle groups. Future studies should include such measurements. Similarly, the expression of tendon properties during MTU function is highly dependent on other limb parameters such as moment arm or muscle architecture (e.g., Scholz et al., 2008; Lee and Piazza, 2009). However, the focus of this study was set on adaptive mechanisms and it is difficult to extrapolate on the impact of the small variations in muscle architecture (no group difference were found in tendon moment arms) without direct measures of MTU behavior. Future research should aim to determine how tendon properties affect MTU behavior, when other biomechanical parameters are accounted for.

## Conclusion

The present results indicate that, by virtue of a lesser hysteresis, long-term exposure to specific loading patterns results in favorable tendon mechanical properties to utilize elastic strain energy. Such an optimization is observed in both the AT and PT and does not seem matched by advantageous MTU morphological features. Collectively, these findings and the theoretical advantage that they imply support the concept of activity-driven adaptations to optimize the utilization of elastic energy. An important future direction will consist in verifying and quantifying more accurately the differences observed here. A longitudinal study, with exhaustive measurement of tendon morphology and/or composition may allow drawing causal inferences from a more compelling data set.

## Author contributions

The experiments were performed by HW, FR, and AK and data were analyzed by HW. HW, FR, AK, EM, and OS designed the work, interpreted the data and wrote the manuscript.

### Conflict of interest statement

The authors declare that the research was conducted in the absence of any commercial or financial relationships that could be construed as a potential conflict of interest.
